# Efficacy of Combined Therapy with Amantadine, Oseltamivir, and Ribavirin In Vivo against Susceptible and Amantadine-Resistant Influenza A Viruses

**DOI:** 10.1371/journal.pone.0031006

**Published:** 2012-01-23

**Authors:** Jack T. Nguyen, Donald F. Smee, Dale L. Barnard, Justin G. Julander, Matthew Gross, Menno D. de Jong, Gregory T. Went

**Affiliations:** 1 Adamas Pharmaceuticals, Inc., Emeryville, California, United States of America; 2 Utah State University, Logan, Utah, United States of America; 3 Department of Medical Microbiology, Academic Medical Center, University of Amsterdam, Amsterdam, The Netherlands; Johns Hopkins University - Bloomberg School of Public Health, United States of America

## Abstract

The limited efficacy of existing antiviral therapies for influenza – coupled with widespread baseline antiviral resistance – highlights the urgent need for more effective therapy. We describe a triple combination antiviral drug (TCAD) regimen composed of amantadine, oseltamivir, and ribavirin that is highly efficacious at reducing mortality and weight loss in mouse models of influenza infection. TCAD therapy was superior to dual and single drug regimens in mice infected with drug-susceptible, low pathogenic A/H5N1 (A/Duck/MN/1525/81) and amantadine-resistant 2009 A/H1N1 influenza (A/California/04/09). Treatment with TCAD afforded >90% survival in mice infected with both viruses, whereas treatment with dual and single drug regimens resulted in 0% to 60% survival. Importantly, amantadine had no activity as monotherapy against the amantadine-resistant virus, but demonstrated dose-dependent protection in combination with oseltamivir and ribavirin, indicative that amantadine's activity had been restored in the context of TCAD therapy. Furthermore, TCAD therapy provided survival benefit when treatment was delayed until 72 hours post-infection, whereas oseltamivir monotherapy was not protective after 24 hours post-infection. These findings demonstrate in vivo efficacy of TCAD therapy and confirm previous reports of the synergy and broad spectrum activity of TCAD therapy against susceptible and resistant influenza strains in vitro.

## Introduction

Antiviral agents are an important therapeutic strategy for adults and children infected with influenza, especially for those hospitalized and at risk for severe illness such as the immunocompromised. While antiviral therapy has been demonstrated to provide some benefit in this patient population [1], [2], [3], [4], the benefit – particularly with neuraminidase inhibitors – is only realized if treatment is initiated within 48 hours of symptom onset, and delaying treatment beyond this time frame is associated with decreased efficacy and greater morbidity and mortality [4], [5], [6]. Furthermore, incomplete suppression of virus replication despite antiviral therapy may result in the emergence of resistance, which is correlated with high and prolonged viral replication such as infection in immunocompromised patients [7], [8], infections with highly pathogenic avian A/H5N1 viruses [9], [10] or primary infection in young children [11], [12], [13]. In addition to treatment-induced resistance, widespread baseline antiviral resistance in circulating influenza virus strains further jeopardizes the effectiveness of existing antiviral drugs. Virtually all influenza A viruses circulating among humans at present are resistant to the adamantanes (amantadine, rimantadine) [14], while seasonal A/H1N1 viruses circulating immediately before the 2009 pandemic were all resistant to the neuraminidase inhibitor oseltamivir [15]. Furthermore, oseltamivir resistance may emerge during treatment, resulting in dual resistance in currently circulating adamantane-resistant viruses [16], [17]. Thus, there is an unmet need for new treatment regimens that can provide greater clinical benefit to those at highest risk of severe disease, and that can reduce the risk of resistance development [7], [8], [9], [10], [11], [12], [13], [14], [15].

Given the limited therapeutic options at present, we chose to optimize the use of available antivirals and to evaluate the effectiveness of a triple combination antiviral drug (TCAD) regimen consisting of amantadine (AMT), oseltamivir (OSL), and ribavirin (RBV). We hypothesized that a combination of drugs acting at different stages in the viral replication cycle might result in synergistic antiviral activity. In earlier studies, we showed that these drugs did indeed act synergistically *in vitro* against drug susceptible viruses, with the triple combination showing greater synergy than any of the double combinations evaluated [18]. In subsequent studies, we found that TCAD also demonstrated synergistic activity against AMT-resistant and OSL-resistant influenza viruses [19]. AMT and OSL clearly contributed to the synergy of the TCAD regimen at concentrations that were clinically achievable and where these drugs had no activity as single agents [19]. In the current study, we extend this work to explore the *in vivo* efficacy and synergy of TCAD therapy in mice. We found that TCAD therapy provided enhanced survival benefit and reduced maximum body weight loss relative to all double combinations in mice infected with fully susceptible, low pathogenic influenza A/H5N1 and AMT-resistant 2009 A/H1N1 viruses. Importantly, the activity of AMT was restored in the context of the TCAD regimen against AMT-resistant influenza strains, confirming earlier *in vitro* data [19]. These data demonstrate the potential of TCAD therapy as a promising, much needed approach to address the dual issues of limited efficacy and antiviral resistance in the treatment of influenza infection.

## Results

### Efficacy of TCAD in mice infected with wild-type and AMT-resistant influenza

**Figure 1:**
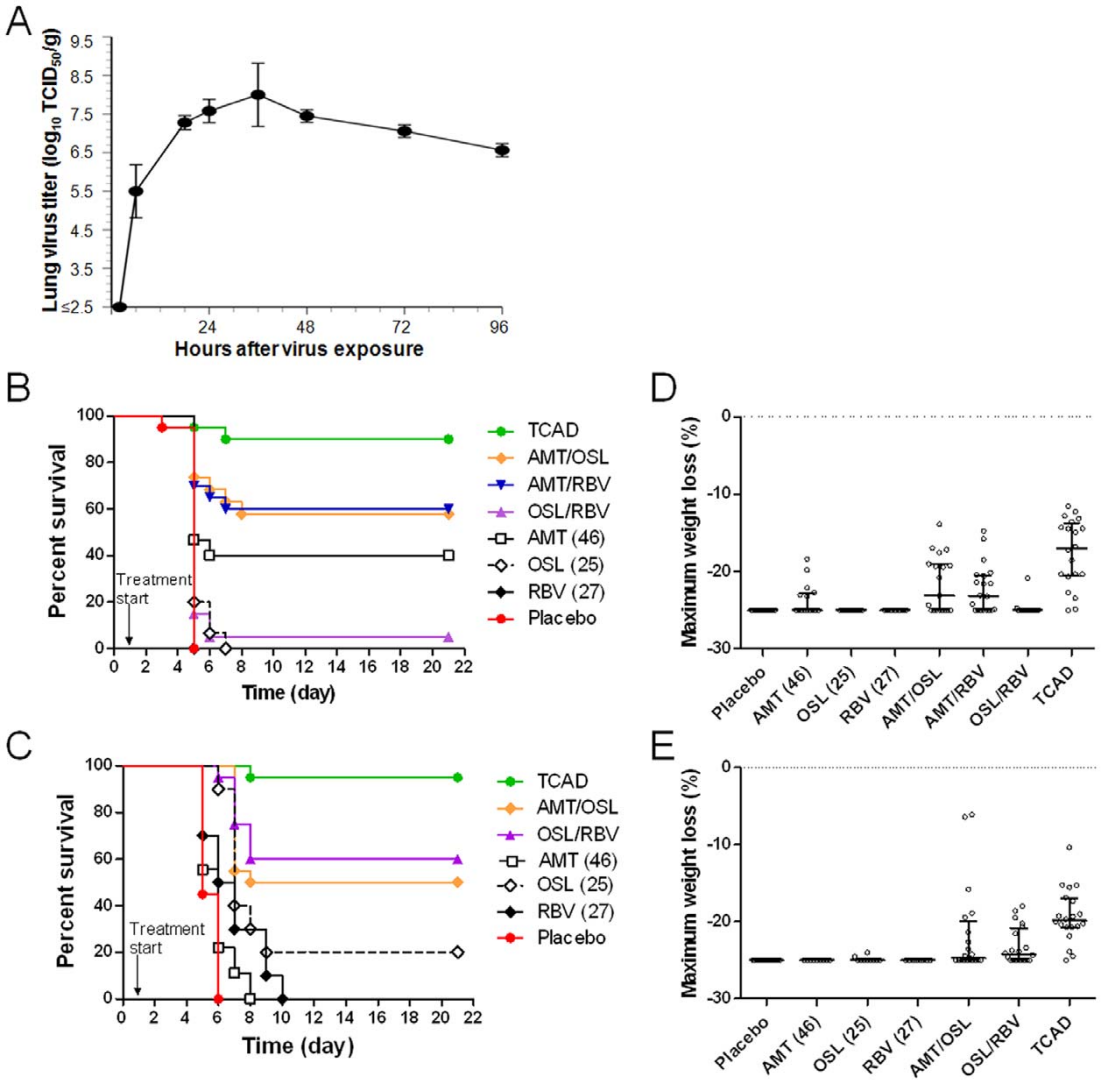
TCAD therapy is highly efficacious in mice. (A) The kinetics of A/H5N1 virus replication in mouse lungs. Mice were infected with an LD_100_ dose of virus and the lungs were harvested at the indicated time points (N = 5), homogenized, and the virus titer was determined by endpoint titration in Madin-Darby canine kidney cells. Kaplan Meier survival curves for treatment of mice infected with lethal doses of (B) a drug susceptible A/H5N1 influenza virus (A/Duck/MN/1525/81) and (C) AMT-resistant 2009 A/H1N1 influenza virus (A/California/04/09). Maximum weight loss analysis for the treatment of mice infected with (D) drug susceptible A/H5N1 and (E) AMT-resistant 2009 A/H1N1. For this experiment, mice were treated with AMT (46 mg/kg/day), OSL (25 mg/kg/day), and RBV (27 mg/kg/day) as monotherapies and in double or triple combinations at the same doses. Treatments were given three times a day for 5 days starting 24 hours after virus challenge, and survival and body weight loss were monitored over 21 days.

To evaluate the efficacy of TCAD in mice, we first optimized the experimental parameters to recapitulate drug exposure and timing of treatment in humans as closely as possible. To determine the appropriate time point for drug administration in treatment studies, the kinetics of influenza virus replication in mouse lungs was determined using the low pathogenic A/H5N1 virus (A/Duck/MN/1525/81). A time course of the virus titer in mouse lungs demonstrated that peak titer occurred 24 hours after exposure (Fig. 1A), indicative this time point was the appropriate trigger for intervention. Next, we determined the drug regimens in mice that would produce plasma exposures comparable to humans. Pharmacokinetic data from single dose administration of AMT, OSL, and RBV in humans and mice were obtained and used to simulate the dosing regimens used in clinical studies (Information S1). Due to species differences in the half-lives of all three drugs, it is not possible to match peak (C_max_) and trough (C_min_) plasma concentrations, but we were able to closely approximate the area under the curve (AUC) and average concentrations (Information S1). Based on the simulations, dosing regimens of 46 mg/kg/day AMT, 25 mg/kg/day OSL, and 27 mg/kg/day RBV (all given in equal divided doses three time daily) were determined to be the clinically relevant doses and were used for murine efficacy studies.

To assess the relative potency of TCAD versus double- and single-therapy, we investigated the survival benefit of TCAD and double combinations and monotherapy in mice infected with drug-susceptible A/H5N1 and AMT-resistant 2009 A/H1N1 (A/California/04/09) influenza viruses. Placebo-treated mice infected with either virus (N = 20) all died or were sacrificed when they reached ≥25% body weight loss (Fig. 1B and 1C). Eighteen of 20 (90%) A/H5N1-infected animals treated with TCAD survived (Fig. 1B), which represented a survival benefit compared to treatment with AMT/OSL (P = 0.05), AMT/RBV (P = 0.055), and OSL/RBV (P<0.001) double combinations (Table 1). Similarly, of mice infected with AMT-resistant 2009 A/H1N1 virus, 19 of 20 (95%) animals treated with TCAD survived (Fig. 1C), which was a significant enhancement in survival benefit relative to OSL/RBV and AMT/OSL (P<0.035, Table 2).

**Table 1:** Statistical comparison of Kaplan-Meier survival curves for treatment of mice infected with wild type A/H5N1 influenza.

Regimen (mg/kg/day)	Survivors/Total	P value vs Placebo	P value vs AMT	P value vs OSL	P value vs RBV	P value vs AMT/OSL	P value vs AMT/RBV	P value vs OSL/RBV
Placebo	0/20	-	-	-	-	-	-	-
AMT (46)	6/15	0.038	-	-	-	-	-	-
OSL (25)	0/15	0.675	-	-	-	-	-	-
RBV (27)	0/14	0.627	-	-	-	-	-	-
AMT (46)/OSL (25)	11/19	0.002	0.305	0.007	-	-	-	-
AMT (46)/RBV (27)	12/20	0.001	0.274	-	0.008	-	-	-
OSL (25)/RBV (27)	1/20	0.583	-	0.925	0.985	-	-	-
AMT (46)/OSL (25)/RBV (27)	18/20	<0.001	-	-	-	0.050	0.055	<0.001

(-) No statistical comparison performed.

**Table 2:** Statistical comparison of Kaplan-Meier survival curves for treatment of mice infected with AMT-resistant 2009 A/H1N1 influenza.

Regimen (mg/kg/day)	Survivors/Total	P value vs Placebo	P value vs AMT	P value vs OSL	P value vs RBV	P value vs AMT/OSL	P value vs OSL/RBV
Placebo	0/20	-	-	-	-	-	-
AMT (46)	0/9	0.090	-	-	-	-	-
OSL (25)	2/10	<0.001	-	-	-	-	-
RBV (27)	0/10	0.002	-	-	-	-	-
AMT (46)/OSL (25)	10/20	<0.001	<0.001	0.185	-	-	-
OSL (25)/RBV (27)	12/20	<0.001	-	0.071	0.001	-	-
AMT (46)/OSL (25)/RBV (27)	19/20	<0.001	-	-	-	0.017	0.035

(-) No statistical comparison performed.

The efficacy of TCAD in reducing body weight loss during infection with A/H5N1 or AMT-resistant 2009 A/H1N1 influenza viruses was also examined (Fig. 1D and 1E). Among A/H5N1-infected mice, significant protection of maximum weight loss relative to placebo was observed in those treated with AMT/RBV or AMT/OSL combinations, but not in those receiving combined OSL/RBV (Table 3). None of the monotherapy regimens significantly reduced maximum weight loss. Greatest protection against weight loss was observed in mice treated with TCAD (P<0.001 compared to all double combinations, Table 3). Against the AMT-resistant 2009 A/H1N1 virus, no significant impact on maximum body weight loss was observed in mice receiving monotherapy with AMT, OSL, or RBV relative to placebo, while significant protection from weight loss was observed for AMT/OSL but not OSL/RBV combinations (Table 4). Greatest protection was seen in animals treated with TCAD, which was significant compared to the AMT/OSL (P = 0.019) and OSL/RBV (P<0.001) double combinations. The percent weight loss as a function of time for surviving mice infected with both viruses and treated with the different antiviral regimens are provided in Figures S1 and S2. The curves show a general trend towards greater protection with the TCAD regimen compared to double combinations. However, since the mice that die or were sacrificed when they reached 25% weight loss were excluded, and thus the number of mice in each group varied as a function of time, no statistical analyses were performed.

**Table 3:** Statistical comparison of percent maximum weight loss for treatment of mice infected with wild-type A/H5N1 influenza.

Regimen (mg/kg/day)	Mean Maximum Weight Loss (%)	P value vs Placebo	P value vs AMT	P value vs OSL	P value vs RBV	P value vs AMT/OSL	P value vs AMT/RBV	P value vs OSL/RBV
Placebo	−25.00±0.06	-	-	-	-	-	-	-
AMT (46)	−23.62±2.12	0.117	-	-	-	-	-	-
OSL (25)	−25.00±0.00	0.988	-	-	-	-	-	-
RBV (27)	−24.97±0.83	0.984	-	-	-	-	-	-
AMT (46)/OSL (25)	−21.62±3.67	<0.001	0.024	<0.001	-	-	-	-
AMT (46)/RBV (27)	−22.25±3.16	0.117	0.117	-	0.002	-	-	-
OSL (25)/RBV (27)	−24.78±0.93	0.794	-	0.797	0.825	-	-	-
AMT (46)/OSL (25)/RBV (27)	−17.58±4.38	<0.001	-	-	-	<0.001	<0.001	<0.001

(-) No statistical comparison performed.

**Table 4:** Statistical comparison of percent maximum weight loss for treatment of mice infected with AMT-resistant 2009 A/H1N1 influenza.

Regimen (mg/kg/day)	Mean Maximum Weight Loss (%)	P value vs Placebo	P value vs AMT	P value vs OSL	P value vs RBV	P value vs AMT/OSL	P value vs OSL/RBV
Placebo	−25.00±0.00	-	-	-	-	-	-
AMT (46)	−25.00±0.00	1.000	-	-	-	-	-
OSL (25)	−24.85±0.34	0.902	-	-	-	-	-
RBV (27)	−25.00±0.00	1.000	-	-	-	-	-
AMT (46)/OSL (25)	−21.63±5.87	<0.001	0.009	0.009	-	-	-
OSL (25)/RBV (27)	−23.11±2.42	0.060	-	0.155	0.123	-	-
AMT (46)/OSL (25)/RBV (27)	−19.27±3.50	<0.001	-	-	-	0.019	<0.001

(-) No statistical comparison performed.

### Effectiveness of delayed treatment with TCAD

**Figure 2:**
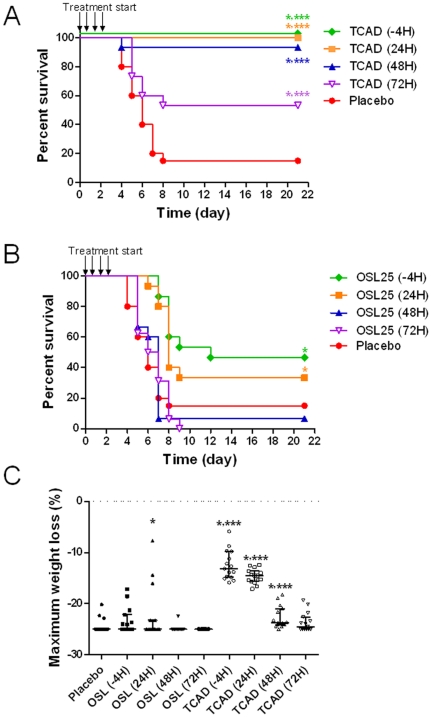
The effects of delayed treatment in mice. Mice were infected with A/H5N1 and treated with (A) TCAD therapy or (B) OSL monotherapy. Treatments were given three times a day for 5 days starting at -4 (prophylaxis), 24, 48, or 72 hours after virus challenge, and survival and (C) maximum body weight loss were monitored over 21 days. Mice were treated with OSL (25 mg/kg/day) alone, or TCAD [AMT (46 mg/kg/day), OSL (25 mg/kg/day), and RBV (27 mg/kg/day)]. *P<0.05 versus placebo; *** P<0.05 versus OSL at the same treatment time point.

To assess the time dependence of the therapeutic benefit, we examined the efficacy of delayed treatment in a lethal A/H5N1 mouse model by comparing survival in mice treated with TCAD or OSL monotherapy at 4 hours pre-infection (-4 hours), or 24, 48, and 72 hours post infection. We found that TCAD was strongly protective when administered up to 48 hours post-infection, with survival rates of 100% when treatment was initiated -4 hours or 24 hours after infection, and 93% when treatment was begun 48 hours post infection (Fig. 2A). Partial protection was provided when TCAD was administered 72 hours after infection (53% survival). In contrast, OSL monotherapy was partially protective only when initiated at -4 hours (47% survival) and 24 hours post-infection (33% survival), and provided no survival benefit when treatment was delayed to 48 or 72 hours after infection (Fig. 2B). TCAD provided significantly greater protection at all time points when compared to placebo or OSL monotherapy (P<0.011 and P<0.008, respectively). Similar trends were observed when maximum weight loss was evaluated as an endpoint (Fig. 2C). Treatment with TCAD starting at -4 hours and 24 hours post infection yielded strong protection from weight loss compared to both placebo and OSL at the same time points (P<0.001). While a smaller benefit was observed when administered at 48 hours post infection, TCAD therapy nevertheless provided greater benefit than both placebo and OSL (P<0.032). The percent weight loss as a function of time for surviving mice treated with TCAD or OSL at different time points relative to infection are provided in Figure S3. Again, while the curves show a general trend towards greater protection with the TCAD regimen compared to OSL or placebo, especially with early drug administration, no statistical analyses were performed due to the fact that the number of mice in each group varied as a function of time due to death.

### Synergy of amantadine, oseltamivir, and ribavirin in vivo

**Figure 3:**
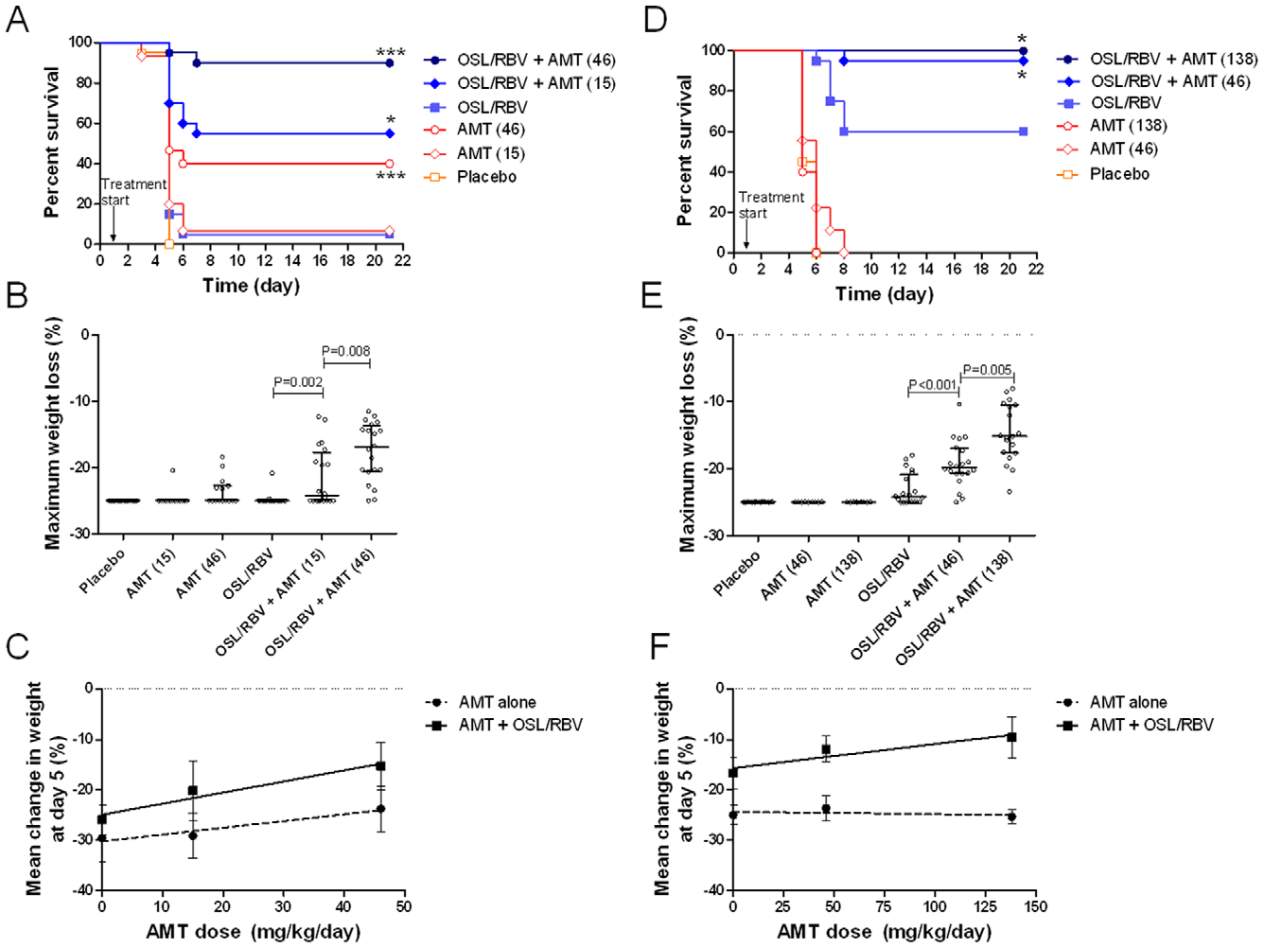
The activity of AMT is enhanced in combination with OSL and RBV against drug susceptible and resistant viruses. (A,B,C) Mice infected with AMT-susceptible A/H5N1 were treated with escalating doses of AMT (0, 15, and 46 mg/kg/day) either as a single agent or in the context of TCAD therapy (with 25 mg/kg/day OSL and 27 mg/kg/day RBV). (D,E,F) Mice infected with AMT-resistant 2009 A/H1N1 were treated with escalating doses of AMT (0, 46, and 138 mg/kg/day) either as a single agent or in the context of TCAD therapy. Kaplan-Meier survival curves for (A) A/H5N1 or (D) 2009 A/H1N1 infection. *P<0.05 versus no AMT, ***P<0.05 versus low dose AMT. Distribution of maximum body weight loss over the course of infection for (B) A/H5N1 influenza or (E) 2009 A/H1N1 influenza. Data bars represent median +/− interquartile range. The mean percentage weight change at day 5 in mice infected with lethal doses of (C) A/H5N1 influenza and (F) 2009 A/H1N1 influenza. Data bars represent mean +/− standard deviation.

To evaluate whether the synergy of TCAD seen *in vitro* is maintained *in vivo*, we examined the dose response of AMT as a single agent and in combination with fixed doses of OSL and RBV in mice infected with either viruses. AMT was dosed at 15 and 46 mg/kg/day against AMT-susceptible A/H5N1, and at 46 and 138 mg/kg/day against AMT-resistant 2009 A/H1N1. Against A/H5N1, AMT at 15 mg/kg/day as monotherapy was not effective at preventing mortality or maximum weight loss (Fig. 3A and 3B), whereas the clinically relevant dose of 46 mg/kg/day resulted in a significant enhancement in survival benefit but had no effect on weight loss. However, in combination with OSL/RBV, treatment with AMT resulted in a dose-dependent increase in survival benefit and inhibition of maximum weight loss, with the 15 mg/kg/day dose producing significant effects compared to OSL/RBV alone, and the 46 mg/kg/day dose producing significant effects compared to the low dose (Fig. 3A and 3B).

Against the AMT-resistant A/H1N1 virus, AMT monotherapy had no impact on survival at 46 or 138 mg/kg/day (Fig. 3D). By comparison, the addition of AMT at 46 mg/kg/day to clinically relevant dose levels of OSL and RBV resulted in significantly greater survival compared to treatment with OSL/RBV alone (19 of 20 versus12 of 20 mice respectively; P<0.001). Increasing the AMT dose to 138 mg/kg/day in combination with OSL and RBV resulted in survival of all treated mice (20 of 20 mice). Similar dose-dependent effects on maximum weight loss were observed (Fig. 3E). Relative to placebo, AMT monotherapy at 46 or 138 mg/kg/day had no effect on weight loss. In contrast, the addition of AMT at 46 mg/kg/day to OSL/RBV resulted in a significantly higher level of inhibition than in mice treated with OSL/RBV alone (P<0.001). Increasing the AMT dose to 138 mg/kg/day in combination with OSL/RBV resulted in a further reduction in maximum weight loss, which was significantly greater than in mice treated with OSL/RBV (P<0.001) or with OSL/RBV in combination with 46 mg/kg/day AMT (P = 0.005) (Fig. 3E). These data demonstrate that AMT was efficacious in combination with OSL/RBV against the AMT-resistant virus at doses which had no effect as monotherapy.

To further quantify the degree of interaction/synergy, the percent weight change at day 5 post-infection was used as an endpoint. The percent weight change at day 5 was used as this represents the latest time point post infection in which most of the mice in the placebo group were still alive, such that a linear dose response model could be created that is not limited by the binary nature of survival. Against A/H5N1, AMT treatment was associated with a dose-dependent reduction of percent weight change at day 5 both as monotherapy and in combination with OSL/RBV (Fig. 3C), with each dose increase producing a significant protection from weight loss (Table 5). Importantly, the slope of the dose response for AMT in combination with OSL/RBV was 1.8-fold greater than the dose response of AMT as a single agent (P = 0.058, Table 5), indicative of synergy. In mice infected with AMT-resistant 2009 H1N1 virus, AMT produced no effect on weight change at any dose as a single agent (Fig. 3F) and the dose response slope was not different from zero (P = 0.678, Table 5). In contrast, AMT in combination with OSL/RBV produced a dose-dependent reduction of percent weight change at day 5, and each dose increase produced a significant reduction in weight loss (dose effect P value<0.001). The enhanced efficacy of AMT in combination with OSL/RBV is further supported by the observation that the dose response slope for AMT in combination with OSL/RBV was significantly greater than the slope for AMT as a single agent (Table 5), demonstrating the synergy of the three drugs in combination (see Information S1 for a derivation of formulas to detect synergy based on dose effects).

**Table 5:** Slopes of the dose response for AMT as a single agent and in combination with OSL/RBV based on percent weight change at day 5.

Virus	Regimen	Slope Estimate	Standard Error	P-value Versus Zero	P-value Between Curves
Wild-type H5N1	AMT alone	0.1311	0.0335	<0.001	0.058
	AMT with OSL/RBV	0.2203	0.0322	<0.001	
AMT-resistant 2009 H1N1	AMT alone	−0.0024	0.0058	0.678	<0.001
	AMT with OSL/RBV	0.0478	0.0078	<0.001	

## Discussion

The availability of an antiviral therapy that has improved potency over standard of care, has broad-spectrum activity against the majority of influenza A strains regardless of the susceptibility, and that can impede the emergence of *de novo* resistance would be of high clinical utility. The pharmacologic rationale for the development of a triple combination antiviral drug (TCAD) composed of amantadine, oseltamivir, and ribavirin was that each drug would target a different stage in the virus life cycle, thereby maximizing the potential for synergy. In addition, given that virtually all A/H3N2 and 2009 A/H1N1 influenza strains are resistant to amantadine, and, in the 2008–2009 influenza season, all seasonal A/H1N1 strains were resistant to oseltamivir, at least two, and possibly three, drugs in the TCAD therapy will be active against any of these viruses. A number of studies have evaluated double combinations of antivirals *in vitro* and *in vivo* with mixed results of synergy [20], [21], [22], [23], [24], [25], [26], [27], [28], [29]. More recently, we and others have demonstrated that double combinations of neuraminidase inhibitors (oseltamivir, zanamivir, and peramivir) were antagonistic *in vitro* and in humans [19], [30].

Combination treatment with AMT, OSL, and RBV was highly efficacious in preventing weight loss and death in mice infected with drug-susceptible A/H5N1 and AMT-resistant 2009 A/H1N1 influenza viruses. The efficacy of TCAD was superior to treatment with single drugs or any of the dual combinations, and the enhanced potency of AMT combined with OSL and RBV *in vivo* confirmed the synergy detected *in vitro* [18], [19]. While comparisons of the efficacy of TCAD versus dual drug regimens approached but failed to reach statistical significance using mortality as an endpoint in a couple of instances (Table 1), the totality of the data support the conclusion that TCAD is superior to all the double combinations. Using both endpoints of mortality and weight loss, the P-values for all the comparisons between TCAD and the double combinations ranged from 0.055 to <0.001. The dose dependent contribution of AMT to efficacy of TCAD therapy in mice infected with 2009 A/H1N1 virus indicates that AMT activity against drug-resistant virus was restored when combined with OSL and RBV, consistent with *in vitro* studies [19]. Furthermore, the beneficial effects of TCAD therapy against A/H5N1 virus were still observed when treatment was delayed to up to 72 hours after infection. Importantly, the dosing regimens used in our murine studies were designed to produce drug exposures similar to those achieved clinically in humans, and were based on pharmacokinetic data obtained for all three drugs in mice and humans. Based on pharmacokinetic data in mice, we determined from simulation that administration of the drugs three time daily were required in order to approximate the plasma exposures in humans, thereby ensuring predictiveness of the drug response (see Information S1).

While the toxicity of the three-drug combination in mice was not directly ascertained in these studies, we have determined the toxicity of the drugs as single agents and have shown that the drugs do not produce detectable toxicity as single agents at the doses used in these studies (data not shown). Furthermore, the doses of all three drugs used in these studies were 5- to >300-fold below the 50% lethal doses of each individual drug [31], [32], [33]. The fact that at the highest dose of TCAD tested (138 mg/kg AMT, 25 mg/kg OSL, and 27 mg/kg RBV) all mice survived whereas none of the mice in the placebo groups survived (Figure 3), and the fact that all mice in TCAD treated groups gained weight during the course of treatment, clearly indicates that any possible toxic effects did not affect our measures of efficacy (e.g. mortality and weight loss).

In the mouse models of infection utilized for these studies, mortality and weight loss were the primary and secondary endpoints, respectively. The mouse infection model was designed as a lethal model, wherein the inoculum was titrated to produce complete lethality – preceded by significant weight loss – in the untreated group within 5 to 7 days. In this model, mortality and weight loss are robust and reproducible endpoints, which enabled us to obtain statistically significant ‘clinical’ efficacy measures using a manageable number of animals. As such, mortality and weight loss were used as the pre-specified endpoints in the experimental design and statistical analysis plan. For these reasons, measurement of viral titers was not considered in the design of the experiments. While viral lung titers might provide additional information, this would require the design and validation of an alternative non-lethal robust model, i.e. one that mimics viral titers and kinetics of human infection, and the dynamic range, effect size, and variability need to be sufficiently validated for antiviral studies.

The exact mechanism by which the synergy of combined treatment is achieved remains unclear, as is the related mechanism by which AMT activity is restored in TCAD against AMT-resistant 2009 A/H1N1. As AMT and OSL target different viral surface proteins – the M2 ion channel and neuraminidase, respectively – it is conceivable that protein-protein interactions are affected by binding of the two drugs at the same time. Interactions between M2 or NA and hemagglutinin, the receptor-binding surface protein, have been shown to affect susceptibilities to AMT and OSL respectively [34], [35]. The presence of the third drug, RBV, is clearly essential for the synergistic antiviral efficacy of TCAD. RBV, which is licensed for hepatitis C and not influenza infection, is a purine analogue with multiple proposed mechanisms of action, both virus- and host-directed, which are not clearly delineated [36]. For example, RBV greatly enhances the potency of interferon for treatment of hepatitis C virus infections, while having marginal activity as a single agent against this virus [37].

Our data suggest that combined therapy with amantadine, oseltamivir, and ribavirin may be an effective and viable therapeutic option for the treatment of drug susceptible and resistant influenza infection. A large randomized controlled trial (NCT01227967) of TCAD therapy versus oseltamivir is currently ongoing in patients at high risk for serious complications from influenza infection, where there is clearly an unmet need for more effective influenza treatment.

## Materials and Methods

### Viruses

The drug-susceptible avian influenza A/H5N1 virus (A/Duck/MN/1525/81 (H5N1)) was provided by Dr. Robert Webster (St. Jude Children's Research Hospital, Memphis TN). It was passaged once in Madin-Darby canine kidney cells (MDCK, ATCC catalog no. CCL034) and three times in mice. The mouse adapted AMT-resistant pandemic influenza A/H1N1 virus (A/California/04/09 (H1N1)) was provided by Dr. Elena Govorkova (St. Jude Children's Research Hospital). The virus was first passaged in MDCK cells and then grown in embryonated chicken eggs. It was then adapted to mice by 9 sequential passages, and then plaque purified in MDCK cells and amplified in embryonated chicken eggs. The virus was then grown in MDCK cells to prepare viral stocks. Sequence analysis was performed to confirm the presence of the AMT resistance-associated S31N substitution in the M2 channel.

### Antiviral drugs

Amantadine hydrochloride (AMT) was obtained from Moehs Catalana S.L. (Barcelona, Spain). Ribavirin (RBV) was obtained from BASF Pharma (Evionnaz, Switzerland) or Bidachem S.P.A (Italy). Oseltamivir phosphate (OSL) was obtained from Dyna International (Huaian) Co., Ltd. (China) or from Cipla Ltd. (Mumbai, India). All drugs were completely dissolved and administered in sterile water. Double and triple combinations were co-formulated and administered as a single solution. The placebo (sterile water) was administered in parallel with antiviral treatments.

### Murine experiments

All animal experiments in this study were conducted in accordance with the approval of the Institutional Animal Care and Use Committee of Utah State University (approval # 552). The work was performed in the University's AAALAC accredited Laboratory Animal Research Center in accordance with the National Institutes of Health Guide for the Care and Use of Laboratory Animals (Animal Welfare Assurance Number A3801-01).

Six-week-old female BALB/c mice from Charles River Laboratories (Wilmington, MA) were anesthetized by intraperitoneal injection of ketamine and infected intranasally with 50-µl suspension of AMT-resistant A/California/04/09 (H1N1) influenza or 90-µl suspension of susceptible A/Duck/MN/1525/81 (H5N1) influenza virus (see Supporting Information for virus passage history). Each mouse received approximately 1×10^4^ CCID_50_ of virus (4× LD_50_) to achieve 100% lethality. For most experiments, treatments were begun 24 hrs after infection and administered three times a day (TID) for a total of 5 days by oral gavage. For all studies, the clinically relevant dosage of each drug (AMT 46 mg/kg/day, RBV 27 mg/kg/day, OSL 25 mg/kg/day) was used alone and in combination, and was projected from animal pharmacokinetic measurements as described in the Supporting Information to provide plasma exposures in mice similar to those in humans. In addition, a 3-fold lower and higher dose of AMT (15 mg/kg/day and 138 mg/kg/day, respectively) was used alone and in combination for some studies. For the delayed treatment experiment, treatments began 4 hrs pre-infection or 24, 48 or 72 hrs after infection. Mice were monitored for 21 days, and mice showing body weight loss of 25% or more from baseline were sacrificed. For the maximum weight loss analyses, mice that died naturally or were sacrificed when they loss ≥25% body weight were assigned a weight loss of −25%.

### Statistical analysis

The primary endpoint for the mouse studies was survival benefit and the secondary endpoint was the percent change in weight from baseline (maximum and at day 5). The survival distributions were estimated using Kaplan-Meier analysis and pairwise comparisons were made using the Cox proportional hazards regression model. Pairwise comparisons of the maximum percent change in weight and percent change in weight at day 5 were done using a one way analysis of variance (ANOVA) model. To evaluate the interaction of AMT in combination with OSL and RBV, the dose-response relationship for AMT alone was compared to the dose-response relationship for AMT with OSL/RBV using weight change at day 5. Using both sets of data, a regression model with separate intercepts and slopes for AMT and AMT/OSL/RBV was fit. Based on this model, a two-sided t-test was then used to test equality of slopes, where equal slopes corresponds to no interaction, positive slope difference corresponds to the presence of an activity enhancement, and a negative slope corresponds to an activity decrement (see derivation in Information S1). For mice that died before day 5, the weights at day 5 were imputed using weights from previous days to fit a linear regression model with weight as the dependent variable and day as the independent variable. The estimated intercept and slope from this model was used to compute a predicted weight at day 5.

## Supporting Information

Figure S1 **Effects of antiviral treatment on weight loss in surviving mice infected with A/Duck/MN/1525/81 (H5N1).** For this experiment, mice were treated with AMT (46 mg/kg/day), OSL (25 mg/kg/day), and RBV (27 mg/kg/day) as monotherapies and in double or triple combinations at the same doses. Treatments were given three times a day for 5 days starting 24 hours after virus challenge, and survival and body weight loss were monitored over 21 days. (A) Single agents. (B) Combination regimens.
(DOC)

Figure S2 **Effects of antiviral treatment on weight loss in surviving mice infected with A/California/04/09 (H1N1).** For this experiment, mice were treated with AMT (46 mg/kg/day), OSL (25 mg/kg/day), and RBV (27 mg/kg/day) as monotherapies and in double or triple combinations at the same doses. Treatments were given three times a day for 5 days starting 24 hours after virus challenge, and survival and body weight loss were monitored over 21 days. (A) Single agents. (B) Combination regimens.
(DOC)

Figure S3 **Effects of antiviral treatments administered at varying time points on weight loss in surviving mice infected with A/Duck/MN/1525/81 (H5N1).** For this experiment, mice were treated with TCAD [AMT (46 mg/kg/day), OSL (25 mg/kg/day), and RBV (27 mg/kg/day)] or OSL as monotherapy (25 mg/kg/day). Treatments were given three times a day for 5 days starting at the indicated time point relative to virus challenge, and survival and body weight loss were monitored over 21 days. (A) 4 hours pre-infection. (B) 24 hours post-infection. (C) 48 hours post-infection. (D) 72 hours post-infection.
(DOC)

Information S1 Correlation of doses in mice to human exposure, and mathematical derivation of synergy as determined by dose response relationships.
(DOC)
