# Supplementary material for: Efficacy of Combined Therapy with Amantadine, Oseltamivir, and Ribavirin In Vivo against Susceptible and Amantadine-Resistant Influenza A Viruses
Source: PLoS One. 2012 Jan 23;7(1):e31006. doi: 10.1371/journal.pone.0031006 (PMC3264642; doi:10.1371/journal.pone.0031006)
Supplement: Figure S2 — Effects of antiviral treatment on weight loss in surviving mice infected with A/California/04/09 (H1N1). For this experiment, mice were treated with AMT (46 mg/kg/day), OSL (25 mg/kg/day), and RBV (27 mg/kg/day) as monotherapies and in double or triple combinations at the same doses. Treatments were given three times a day for 5 days starting 24 hours after virus challenge, and survival and body weight loss were monitored over 21 days. (A) Single agents. (B) Combination regimens. (DOC) [file pone.0031006.s002.doc]

**Figure S2**

**A**

**B**
