# Supplementary material for: Efficacy of Combined Therapy with Amantadine, Oseltamivir, and Ribavirin In Vivo against Susceptible and Amantadine-Resistant Influenza A Viruses
Source: PLoS One. 2012 Jan 23;7(1):e31006. doi: 10.1371/journal.pone.0031006 (PMC3264642; doi:10.1371/journal.pone.0031006)
Supplement: Figure S3 — Effects of antiviral treatments administered at varying time points on weight loss in surviving mice infected with A/Duck/MN/1525/81 (H5N1). For this experiment, mice were treated with TCAD [AMT (46 mg/kg/day), OSL (25 mg/kg/day), and RBV (27 mg/kg/day)] or OSL as monotherapy (25 mg/kg/day). Treatments were given three times a day for 5 days starting at the indicated time point relative to virus challenge, and survival and body weight loss were monitored over 21 days. (A) 4 hours pre-infection. (B) 24 hours post-infection. (C) 48 hours post-infection. (D) 72 hours post-infection. (DOC) [file pone.0031006.s003.doc]

**Figure S3**

**A B**

**C D**
