# Supplementary material for: Efficacy of Combined Therapy with Amantadine, Oseltamivir, and Ribavirin In Vivo against Susceptible and Amantadine-Resistant Influenza A Viruses
Source: PLoS One. 2012 Jan 23;7(1):e31006. doi: 10.1371/journal.pone.0031006 (PMC3264642; doi:10.1371/journal.pone.0031006)
Supplement: Information S1 — Correlation of doses in mice to human exposure, and mathematical derivation of synergy as determined by dose response relationships. (DOC) [file pone.0031006.s004.doc]

# Supporting INformation

## Correlation of Doses in Mice to Human Exposure

The dosing regimens in mice employed in these studies were determined from the anticipated plasma exposures in humans. An effort was made to match multiple pharmacokinetic parameters across species (C_max_, C_min_, AUC and C_ave_). Due to inherent differences between species in the elimination half-lives of all three drugs, matching all PK parameters is not feasible, therefore the area under the curve (AUC) and average concentration (C_ave_) were chosen as the primary PK parameter of importance. The data from the oral administration of a single dose of 100 mg AMT, 75 mg OSL, and 600 mg RBV in healthy volunteers (manuscript in preparation) were used to perform simulations for AMT and OSL, and RBV over a 5-day treatment period, using a noncompartmental model in WinNonLin software version 5.0.1. In addition, data from the oral administration of single dose of 1, 10, and 100 mg/kg AMT, OSL, and RBV in mice were used to model TID dosing (three times daily, dosing regimen utilized in mouse studies) to determine the mouse equivalent dose for efficacy studies.

The human drug regimens used for modeling were based on the drug regimens used in a pilot TCAD studies in immunocompromised patients: 75 mg AMT TID, 50 mg OSL TID, and 200 mg RBV TID (clinicaltrials.gov identifier NCT00867139, manuscript in preparation). For all three drugs, the C_ave_ was calculated as AUC_0-120_/120 hours for comparison to mice. The C_ave_ in humans for AMT, OSL, and RBV were calculated to be 436, 283, and 464 ng/mL, respectively. The average exposure in mice (C_ave_ calculated as AUC_0-120_ /120 hours), for AMT 46 mg/kg/day (15.3 mg/kg TID), OSL 25 mg/kg/day (8.3 mg/kg TID) and RBV 27 mg/kg/day (9 mg/kg TID) were calculated to be 417, 255, and 457 ng/mL, respectively, which closely approximates the human exposures. The lower 15 mg/kg/day (5 mg/kg TID) and higher 138 mg/kg/day (46 mg/kg TID) doses of AMT utilized in these studies are anticipated to produce exposures roughly 3-fold below and above average human exposures, respectively.

## Mathematical Derivation of Synergy as Determined by Dose Response Relationships

To evaluate the interaction of AMT in combination with OSL and RBV, the dose-response relationship for AMT alone was compared to the dose-response relationship for AMT with OSL/RBV.  Using both sets of data, a regression model with separate intercepts and slopes for the AMT and AMT/OSL/RBV experimental data was fit.

The following expression for the activity of each component to the fractional weight loss, W, assuming the dissimilar site model of Pritchard and Shipman and Chou and Talalay is:

$$(1)$$

where:

C_AMT_ – concentration of AMT; with a= to the linear dose response term

C_OSL_ – concentration of OSL; with b= to the linear dose response term

C_RBV_ – concentration of AMT; with c= to the linear dose response term

X(C_AMT_ ) – fractional inhibition of amantadine as a function of C_AMT_

Y(C_OSL_) – fractional inhibition of amantadine as a function of C_OSL_

Z(C_RBV_) – fractional inhibition of amantadine as a function of C_RBV_

G1(C_AMT_,C_OSL_) – inhibition via two-way interaction of AMT with OSL as a function of C_AMT_ and C_OSL_

G2(C_OSL_,C_RBV_) – inhibition via two-way interaction of RBV with OSL as a function of C_RBV_ and C_OSL_

G3(CAMT,CRBV) – inhibition via two-way interaction of AMT with RBV as a function of C_AMT_ and C_RBV_

H1(C_AMT_,C_OSL_,C_RBV_) – inhibition via three way interaction of all three components as a function of C_AMT_, C_RBV_, and C_OSL_

For the special case of an AMT dose response, we take the partial derivative of both sides, df/dC_AMT_, and setting Y = Y_o_ and Z= Z_o_ yields:

$$\text{(2)}$$

Over a narrow concentration range, we can assume all of these functions are linear, first order equations (i.e, only the first 2 terms in the Taylor series are significant). This leads to two simple expressions for this special case:

For AMT alone:

$$\text{(3)}$$

and integrating gives us

$$\text{(4)}$$

While for AMT in combination with a fixed amount of OSL and RBV:

$$\text{(5)}$$

where g1 = dG1(C_AMT_, C_OSL_ = 0.3 μg/mL)/dC_AMT_

g3 = dG3(C_AMT_, C_RBV_ = 0.6 μg/mL)/dC_AMT_

h1 = dH1(C_AMT_, C_OSL_ = 0.3 μg/mL, C_RBV_ = 0.6 μg/mL)/dC_AMT_

α_o_ = Y_o_ + Z_o_ -Y_o_Z_o_

and integrating (5) gives us:

$$\text{(6)}$$

For the results shown in Figure 3 and Table 1, the fractional contribution of the OSL, RBV double combination to the observed effect is negligible, thus we can set α_o_ to 0. If we now evaluate these data sets for the presence of an interaction, the null hypothesis is:

Evaluation of this null hypothesis produces 3 possible outcomes:

If a + g1 + g3 +h1 > a, then net interaction terms are positive; synergistic

If a + g1 + g3 +h1 = a, then net interaction terms are zero; additive

If a + g1 + g3 +h1 < a, then net interaction terms are negative; antagonistic

A two-sided t test was then used to test equality of slopes. The results are presented in the text of the manuscript. In both studies, the results indicate a positive interaction is present, consistent with the detection of synergy in vitro by applying the same governing formula to a full Latin square analysis.

1. Prichard MN & Shipman C, Jr. (1990) A three-dimensional model to analyze drug-drug interactions. *Antiviral Res* 14(4-5):181-205.

2. Chou TC & Talalay P (1984) Quantitative analysis of dose-effect relationships: the combined effects of multiple drugs or enzyme inhibitors. *Adv Enzyme Regul* 22:27-55.
